# What makes you think you are conscious? An agnosticist manifesto

**DOI:** 10.3389/fnhum.2015.00170

**Published:** 2015-04-07

**Authors:** Cees van Leeuwen

**Affiliations:** ^1^Mind and Brain Research Unit, University of LeuvenLeuven, Belgium; ^2^Center for Cognitive Science, TU KaiserslauternKaiserlautern, Germany

**Keywords:** phenomenal experience, hard problem, personal identity, selfhood, philosophy of mind, theories of consciousness, brain diseases, cognitive neuroscience

## Abstract

The qualitative character of consciousness, its “what-it-is-likeness”, is a contested issue, both in philosophy and psychology. I argue that, rather than by conceptual analyses, the status of “what-it-is-likeness” has to be decided by empirical investigation. Pending the outcome, we should maintain an agnostic stance, in order to remove the bias in favor of fictionalism from our study of consciousness,. I illustrate this with the notion of “ownership unity”. People adhere to the belief of a single, unified self as the owner of their experiences, in spite of abundant dis-unities in the informational content of their experience. On one reading, this supports the notion that the unity of experience is no more than a convenient fiction, based on an illusory experience of unity. Cognitive neuroscience is slanted in favor of such understanding, insofar it emphasizes functional specialization and localization. To restore the balance, I present a complementary perspective: the view that the experience of unity is afforded by the intrinsic, multiscale brain dynamics. This approach offers a biological substrate for unity of experience as a regular scenario within certain boundary conditions, as well mechanisms that may let it go astray.

## Introduction

“Whatever is the lot of humankind

*I want to taste within my deepest self*.

*I want to seize the highest and the lowest*,

*to load its woe and bliss upon my breast*,

and thus expand my single self titanically

and in the end go down with all the rest.”

*—Johann Wolfgang von Goethe, Faust: First Part*^1^

From its inception as a scientific discipline, the main focus of psychology has long been on consciousness (Wundt and Pintner, 1912); this lasted its methods were deemed unscientific by behaviorism. Even though many still recently believed consciousness to lie outside of the scope of scientific explanation (Fodor, 2000) or denied its existence altogether (Dennett, 1992), heralds of a new dawn such as Baars (1988, 1997) have brought it back to the forefront of psychology and related sciences. To date, cognitive neuroscientists study brain states as “correlates” of, mostly what Rosenthal (1986) called transitive consciousness, or consciousness of something, using brain imaging techniques including fMRI, EEG, or MEG, Behavioral and clinical neuroscience study intransitive consciousness (Rosenthal, 1986), offering tools for inducingmild, temporary alterations to conscious states, such as TMS or tDCS, and are investigating more dramatic changes such as anesthesia or coma. Psychophysicists offered methods such as signal detection to study what we are able or unable to consciously detect. Experimental psychologists have contributed methods such as change blindness and attentional blink paradigms, to study “lapses of conscious experience”. This flurry of activity has led to an abundance of experimental results and a major demystification of consciousness.

While the interest in consciousness is widespread, there is the nagging suspicion that our scientific studies leave something out of the equation. When talking about consciousness, we distinguish processes such as visual or auditory recognition and discrimination, sensory experience, reasoning and decision-making, as well as properties we ascribe to them, such as “ownership unity”, “egocentric perspectivity”, agency, contentfulness, but seem to be ignoring its “qualitative character” (Welshon, 2013)—the latter, following Nagel’s famous paper (Nagel, 1974), titled “What is it like to be a bat?” is sometimes being addressed, somewhat tongue-in-cheek, as “what-is-it-likeness”—a term I will adopt here, if only for the sake of reappropriation.

Whereas hardly any of consciousness’ properties are a complete mystery any more, “what-is-it-likeness” remains elusive. Not surprisingly, it is this the opponents of consciousness are most eager to drop from their lexicon. “What-is-it-likeness” is what David Chalmers (1996) called the “hard problem”of consciousness. Here I will discuss whether we have reasons to believe that “what-is-it-likeness” is for real. I will first consider what philosophers have to say about this subject. After concluding that philosophy cannot secure its status, it becomes clear that this a matter of empirical investigation. Here I will observe that even though recent evidence may seem slanted against “what-it-is-likeness”, we are best advised to keep an agnostic stance; after all, “what-is-it-likeness” may have a basis, for instance in the intrinsic dynamics of our brain. I will describe my version of this view in some detail.

## What Philosophers Have to Say

And so I sit, poor silly man

*No wiser now than when I began*.

*—Faust, lines 358–359*

While confronting a mechanical and ultimately physical world, rationalist philosophers have tried to establish “what-is-it-likeness” through intuitive apprehension “by a simple act of mental vision” (Descartes, as cited in Hintikka, 1962), of the cognizing “I”. In Descartes’ famous statement: *cogito ergo sum*, the apprehension of his first person experience is one of self-evident existence. This may be considered a case of what psychologists today know as introspection (which, by the way, may differ from what Wundt had in mind).

Introspective reports are laden with ambiguities. Schwitzgebel (2008) highlights “…emotional experience (for example, is it entirely bodily; does joy have a common, distinctive phenomenological core?), peripheral vision (how broad and stable is the region of visual clarity?), and the phenomenology of thought (does it have a distinctive phenomenology, beyond just imagery and feelings?)”. Any effort to our reports by resolving these ambiguities by reflection is doomed to recede into armchair theorizing. As Graziano (2014) has succinctly put it: “When we introspect and seem to find that ghostly thing—awareness, consciousness, the way green looks or pain feels—our cognitive machinery is accessing internal models and those models are providing information that is wrong. The machinery is computing an elaborate story about a magical-seeming property. And there is no way for the brain to determine through introspection that the story is wrong, because introspection always accesses the same incorrect information.” Indeed, introspection therefore can mislead us in thinking that our experiences have qualitative character. A radical form of this position is taken by the later Wittgenstein, who considers all talk of phenomenal experience a “myth”, that doesn’t stand to public scrutiny.

One might wish to object: likewise as our experience could not have been right about itself without existing, doesn’t the fact that it is in error about itself prove its existence? This, however, is, as Hintikka (1962) points out, is a logical fallacy that is a consequence of misreading Descartes’ famous dictum. The fallacy is of the type: Homer must have either been a Greek or a barbarian. Therefore he must have existed, since if he was a Greek, he must have existed, and if he was a barbarian, he must equally have existed. This flaw occurs because first-order logic typically works with objects that are implied to exist. As with Homer, the existence the existence of our “what-is-it-likeness” is at stake, and can thus not be proven by this syllogism.

Instead, some philosophers have claimed that “what-is-it-likeness” has a unique way of referencing physical properties (Loar, 1990). But that doesn’t stand up against scientific scrutiny. True, psychophysics occasionally produces nice, apparently lawful relationships between physical and sensory quantities. But these are rarely stable across individuals or within individuals over time. Over time the visual system adjusts its sensivity to what is most relevant to observers in their environments (Jurica et al., 2013). This may be called adaptive management of inherently limited resources, or economizing. From the perspective of economizing systems, it could be expected that, for instance, visual experience is often relying on moderately reliable cues. Indeed, in case of dynamic displays, for instance, Anstis (2010) has demonstrated that the visual system takes “ease of perception” as a cue for speed of movement; this observed fact is responsible for a range of motion illusions. What to make of Loar’s program in this light? Economizing most likely is not unique to experiencing subjects; we will find this function up and down the ladder of evolution. Referencing physical objects certainly it isn’t.

Some philosophers might think that the unique way of referencing is reserved for a certain subclass of phenomena, such as pain. According to Kripke (1980), pain is a mere presence; it is what it is to the person. Such an approach might seem to protect the status of the experience, but in fact locks it up in a cocoon of dogmatism. Because this way of referencing is deemed unique to the individual, for a category of observations it becomes ground truth, and hence these observations incorrigible. This would be particularly sad news for a person with phantom limb pain. The person would be told to live with her incorrigible sensation. In fact, corrective therapy exists and can boast success, based on what is known about the plasticity of the sensory-somatic brain map (Ramachandran and Blakeslee, 1998).

To summarize, introspection and sensation are intrinsically unreliable in their way of referencing; even sensations claimed to be mere presences, such as pain are not incorrigible. An emphasis on reference does not offer a privileged status to the qualities of phenomenal experience. This offers them up as candidates for reduction or elimination. Instead, we might gain a more empirically informed understanding by emphasizing their functionality in the context of an economizing system. Such a notion, however, requires giving up all hope of being able to validate their existence in terms of identifiable referents.

Then there are those who, following Husserl, propose a constitutive role for consciousness in our understanding. Georgalis (2006) develops a theory of representation, in which representions are of necessity established through the intention of a conscious being. The intention of the act of referring is also sufficient, according to Georgalis (2006), even if there is nothing in the world to satisfy the intention. This might do for a philosophical theory of representation, but is entirely formal and sterile from a psychological perspective. The phenomenal aspects of the act play no role in the postulated logical necessity of consciousness for establishing representation in the theory. Still, it will offer a valuable illustration of a point I want to make in the next section.

We may conclude that neither reference, nor a first-person analogue to the Fregean concept of *sense* as proposed by Georgalis (2006) can establish a need for real, qualitative phenomenal experience. But perhaps, the role of experience in our cognitive functions could. However, according to Chalmers’ (1996) “philosophical zombie” thought experiment, an individual may exist, at least logically speaking, who functions in all possible respects in exactly the same way as a conscious agent does, but who lacks conscious experience. When questioned, the zombie will claim to have them, falsely but entirely convincingly. In fact, it has to, as this is exactly what its non-zombie counterpart would do.

It is worthwile to mention that the logical possibility of such a zombie does not amount to behaviorism. Behaviorism does not deny the presence of conscious experience, but claims that its constituents are behaviors (partly overt, and partly covert, and some physiology, to account, e.g., for hunger pangs). To behaviorism, the proposed zombie, who behaves in all possible ways like a normal individual would effectively be identical to that individual. In other words, a zombie, according to behaviorism, cannot exist (Kirk, 1999). Behaviorism, however, has other problems. Consider, for instance, an individual with locked-in syndrome: a conscious person bereft of all overt behavior. Behaviorism must either deny that the person is genuine consciousness or revert to the claim that all its components are covert (or physiological), which reduces its claims to a tautology.

The logical possibility of philosophical zombies permits a principled skepticism, firstly, with respect to other minds. This observation gains practical significance in light of our increasingly frequent conversations with dialog bots, which are rapidly becoming more and more convincing. We may be surrounded by bots, or zombies.

The skeptical contagion does not halt at the self. We have seen that our introspections could be misleading; but now even worse, the sum total of our experiences could be delusions. This is hilariously illustrated in a one-page essay by Smullyan (1980/2002). Loosely paraphrased, it runs as follows: A philosopher who can not bear the lightness of being decides, before going to bed, to take a pill that will turn him onto a philosophical zombie, Next morning when he wakes up he exclaims: “Oh s**t! The pill didn’t work!” He has to, either way, hasn’t he? The stubborn certainty about your own first-person experience is something you share with your Zombie counterpart. In the same way it is deceiving others it has to be deceiving itself.

Even if we were all philosophical zombies, this would have no effect whatsoever on our lives, as all of us will continue to act as if we possess “what-is-it-likeness”, and continue deceiving ourselves and each other about having it. The upshot is that it may be a complete epiphenomenon, i.e., it is completely irrelevant towards the course of events in our lives and at worst a complete fiction. Thereby the philosophical zombie argument appears to be cutting through the ties of psychological explanation and “what-it-is-likeness.”

## What Psychologists Have to Say

“When scholars study a thing, they strive to kill it first, if it’s alive; then they have the parts and the’be lost the whole, for the link that’s missing was the living soul.”

*—Johann Wolfgang von Goethe, Faust, Part One*

Based on Chalmers’ zombie thought experiment, we may call “what-is-it-likeness” redundant, or so it seems. But this is true only if one buys into the philosophical hubris of it. Remember the definition of a zombie as an individual in all possible psychological functions identical to a person with, yet having no qualitative conscious experience. It uses “all possible psychological functions” as if it were a well-delineated set. But this could be debated. What delineates this set cannot be decided apriori and is to be determined, based on empirical investigation. Such investigation may determine whether the concept of “psychological zombie” turns out to be meaningful, because it is empirically possible that some functions necessitate qualitative consciousness. And so, as far as “what-is-it likeness” is concerned, the ball is in the court of psychology and its sister sciences.

Can we envisage any psychological functions that might necessitate qualitative consciousness? We have seen that Georgalis (2006) required conscioiusness for representation, but did not involve its qualitative character. We might, however, image his definition of representation embedded in the context of a psychological theory. For instance, I exclaim: “Oh what a beautiful flower!”. According to Georgalis, this constitutes representation, because it results from an intentional act to refer by a conscious individual. This, even though the flower may exist only in my imagination. But if so, what causes such an expression in a conscious being as such, psychologists would ask. If the answer is: it must be the quality of the image as experienced, we have just created a theory of psychological function in which “what-is-it-likeness” plays an ineliminable role. This is because it is a formal requirent for representation, which renders it immune to the zombie thought experiment.

Should we endorse such a theory, the ball would be back in the philosophers’ court, as the question would now be how “what-it-is-likeness” can exist in a physical world. Psychologists, however, even those who are in the consciousness business, are somewhat reluctant to endorse talk of “what it is likeness”. The problem, however, is, it is closely intertwined with most, if not all, psychological functions and properties. How we experience a color depends on the object the color belongs to, and the context the object is embedded in McCann et al. (2014), including our beliefs about the object and its context. Think of how hard it is to detect incongruent playing cards, e.g., a red five of spades or black 7 of hearts. According to Welshon (2013): “… part of what conscious experience is like for me is that it is from an egocentric perspective and unified as mine; part of what conscious experience is like is that it has qualitative character; part of what conscious experience is like is that it is or seems to be about something; and part of what conscious experience of myself is like is that I am an agent” (p. 841). Welshon observes that this interwovenness easily leads to conflation and that “what-it-is-likeness” should therefore best be left untouched.

But rather, one could argue, this interwovenness underscores how central a role “what-it-is-likeness” play in how we understand ourselves in our everyday lives. This remains true, even if we are all zombies. We might therefore equally proclaim that understanding “what it is likeness” is the ultimate goal of studying consciousness.

The interwovenness with so many of our psychological functions could even be considered a defining characteristic of consciousness. According to Baars (1988, 1997), consciousness is a collection of functions that have global access to, and provide input for, various more specialized processing systems. Together, they make up the global workspace of the eponymous theory. The resource, or resources, these functions share, are likely to be are strongly interconnected set of components that, as a system, could enable us to experience “what-it-is-likedness”.

Perhaps another reason for reluctance in addressing “what-it-is-likeness” is the discrepancy, highlighted by philosophers, between private certainty (Descartes) and public inaccessibility (Wittgenstein). We have seen the philosophical match between them to be undecided.Yet, researchers feel that they may have to take sides with one or the other. we see phenomenological doctrines making private certainty the core of their theoretical commitment vs. mainstream psychology, which seems to have sided with the accessible.

But exactly because the minds have become so divided on this issue, an unbiased contribution from science is desparately wanting. Rather than taking sides, we should practice *agnosticism* with respect to the ontological status of “what-is-it-likeness”. Making progress requires suspending judgment. For science to have the last word, we should proceed in understanding consciousness through empirical research, without having in advance either to affirm it as truth or reject it it as myth.

I will argue that our current knowledge of consciousness does not decide the status of “what-is-it-likeness”. In particular, I will illustrate this with the property of ownership unity, i.e., the sense that you possess a self that owns your experiences, is of special interest in this debate, because people hold on to ownership unity despite what appears overwhelming scientific evidence to the contrary. The experience of unity, or what someone calls “self”, may be no more than the product of a number of discrete psychological functions. This might suggest that the experience of unity itself is unreal. I will argue that such a conclusion would be premature: even if the unity of experience is a figment of our imagination, the resilience of of ownership unity may be still have a real basis. I will argue that the basis may exist in the biology of our brain.

## What Unity is There in Consciousness, Really?

In me there are two souls, alas, and their

*Division tears my life in two*.

One loves the world, it clutches her, it binds

*Itself to her, clinging with furious lust*;

The other longs to soar beyond the dust

*Into the realm of high ancestral minds*.

*—Faust, lines 1110–1115*

Some functions that involve conscious experiences, including imagery, predominantly have sensory components, some involve our willful actions and thus appear linked to our motoric abilities, still others appear linguistic or amodal in nature. What binds them together is the tendency of the subject to identify with them: these are my thoughts, my sensations, etc. This is what we experience, and have come to describe, as our *self*.

Why do we see ourselves as inhabited by a single the arbiter of our experience and chief commander of our actions? In a religiously motivated perspective, unity is provided by the concept of a soul. The precise relation between soul and self may vary according to religious persuasion; in a Christian context the divine aspects of the soul pose certain norms which the self must reflect. As agnosticists, however, we need not take such beliefs for granted. We may wonder: why is it one rather than two, or 20; or, perhaps, wouldn’t a committee do just as well? According to Dennett (1992), the self is just a fictional center in a multitude of narratives that convey our experience. The mere convenience of a fiction does not explain why we adhere to ownership unity with such great stubbornness.

The principle that we perceive ourselves as one is hardly ever violated, except in some cases of extremely dissociative identity disorder (often a result of severe psychological trauma involving prolonged abuse and/or neglect in infancy). Extremely dissociative identity disorder patients show multiple alters, each of which may claim ownership unity for part of a person’s experience and biography. These phenomena may well be controversial. But it is exactly the kinds of controversies (e.g., whether these personalities genuinely experience these alters or are they merely deluding themselves and others) that the agnosticist view has no stake in. What matters is whether they show the symptoms, have a history that could explain them, and whether they have a biological basis. For this, see (Perry, 2002; Teicher, 2002).

We may wish to distinguish between the self as a persistent unity and the unity of the momentary experience of selfhood. Dissociative personalities may lack persistent unity but each of their alters may still have a unified sense of self at a moment-to-moment basis or, what Stroud (1967) calls the “psychological present”. Theorists have suggested that such a minimal sense of unity of the self may be necessary to keep “the ongoing process of tracking and controlling global bodily properties” (Blanke and Mentzinger, 2009, p. 8) and may, therefore, derive from these processes. According to Welshon (2013), perception, imagery, interoception and proprioception might also play a role in establishing such a minimal sense of self.

No matter how richly or sparsely endowed, the minimal self is not indispensable for having a persistent self (or maximal) sense of self. Welshon (2013) points to patients with Balint syndrome. These patients have simultagnosia, a form of visual feature binding agnosia, who experience events in the world as a set of discrete snapshots. These phenomena are not uncontested, but again, from an agnostic point of view we needn’t care. In spite of the moment-to-moment discontinuities, these patients feel they own their experiences. Most already had a fully developed sense of ownership unity before the lesion that led to their symptoms; Balint syndrome is rarely reported in children. Drummond and Dutton (2007), however, report a patient with these symptoms as a congential condition.

The distinction between unity in the persistent and the minimal sense of self refers to the spatial and temporal scale at which unity is experienced. Based on the double dissociation between, on the one hand, extreme dissociative personalities (who have minimal but lack maximal selves) and Balint syndrome patients (who have maximal but not minimal selves), I propose that both may the product of distinct biological mechanisms.

A further distinction is between ownership and content unity (Welshon, 2013) In some epileptic patients, the corpus callosum and other connectors such as the anterior commissure and hippocampal commissure are cut through to reduce the severity of seizures. This means there is hardly any communication possible between the left and the right hemisphere. Meyers, Sperry and Gazzaniga started to pursue the question how the thus separated hemispheres function (Gazzaniga et al., 1962). Among other things, these patients cannot name a familiar object they are allowed to touch but not able to see, when it is presented to their left hand. This is because they sense the object in the right hemisphere, whereas their speech is located in the right hemisphere. Despite their impairments, callosal patients function normally under most circumstances. Callosal patients still experience themselves as a single individual (Gazzaniga, 1989); they have a perfectly normal sense of ownership unity.

Schiffer et al. (1998) speculate that nevertheless, two separate selves inhabit the hemispheres—but that the latter of these is dominant. This, however, may appear too fanciful. According to Gazzaniga (1989), the left hemisphere has the role of interpreting the available information in terms of a self. This would imply that the self is a narrative construction, in accordance with Dennett (1992).

We could, however, maintain that the psychological ownership unity is jointly maintained by the two hemispheres even in cases where direct communication between them is reduced. Most of these patients, after all, already had a unitary self before they were lesioned. One notable case of a patient who had the lesion congenitally was Kim Peek (Treffert and Christensen, 2006). His may be a case of ownership unity that is not biologically, but *informationally* based. In most normal situations both hemispheres of split brain patients receive plenty of same information through their senses. Foveal vision goes to both hemispheres, most sounds are binaural, etc. Kim Peek, however, was unusual in many respects; he had savant syndrome, and could read two pages at the same time. His brain had language centers in both hemispheres as well as other anomalies. These anomalies might be strong enough to consider the possibility that also his sense of self was different from ours.

**Figure 1:**
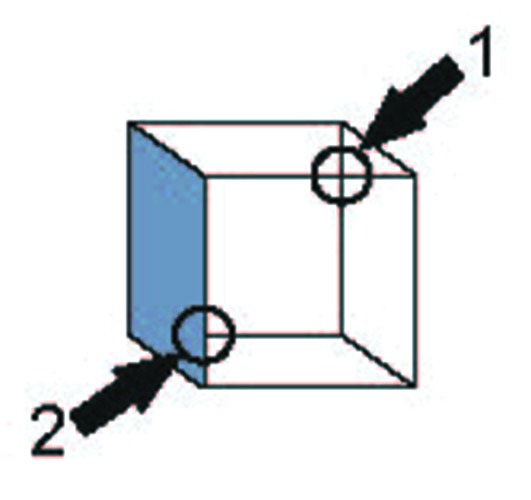
**Ambiguity in the Necker cube is not constrained by local bias**. Circles indicated by arrows represent alternative areas to which a prior instruction draws attention. No arrows or circles were present in the actual display. Area 1 contains an X-junction indicating an ambiguous cube; Area 2 contains a T-junction which yields a strong bias to the orientation of the cube. When attention is drawn to Area 1, the cube reverses despite the presence of a local disambiguation in Area 2. Adapted from van Leeuwen and Smit (2012) after Peterson and Hochberg (1983).

Whereas Welshon does not distinguish minimal sense of self from ownership unity. However, I would like to distinguish the two as in the laboratory of the psychologist, content unity failure is commonly encountered with individuals who have a perfectly normal (minimal and maximal) sense of self. Peterson et al. used Necker cubes that were locally disambiguated (Figure 1). When the disambiguated area is overtly (Peterson and Hochberg, 1983) or covertly (Peterson and Gibson, 1991) attended to, observers perceive the orientation according to the local disambiguation. But when another, unbiased area is attended to, the disambiguation fails to influence the perceived orientation. Information of the parts, therefore, is only loosely integrated with each other. Nevertheless, we *experience* the cube as a unified whole. The minimal unity of the self has no basis in the available visual content.

As I argued elsewhere (van Leeuwen, 2007), this phenomenon has a biological basis; it is readily explained by the neurodynamics of the visual system. The conditions in which it arises do not hold in general, but are restricted to presentation in isolation, in combination with prolonged focused attention to part of the figure. This results in a lack of global coherence in the brain state, that prevents information from becoming integrated. Under slightly more ecologically valid conditions, involving the presence of a flanking context, these modes still exist, but alternate with others that enable global integration (van Leeuwen and Smit, 2012).

Whereas in Peterson’s studies participants keep their eyes fixated on a single spot, in free viewing we fixate on certain locations for 200–300 ms, after which our eyes move rapidly to the next location by means of a saccade. Nevertheless, we experience the external world as continuous across saccades. We are usually not even aware of our eye-movements in exploring a scene. Neither are we usually aware of our eyes blinking. In other words, our minimal sense of self ignores the discontinuities in the available visual content.

These phenomena, too, have their basis in the biology of the visual system. Saccadic (and blink) suppression mechanisms ensure that during saccadic eye-movements, the signal from the eye is interrupted and cannot alert us to the change in eye-position. Prior to the eye-movements, a shift in attention takes place that offsets the selectivity in the receptive fields to remain maximally position-invariant. It is clear, therefore, that these mechanisms have adaptive significance. Without them, our perceptual information would constantly suffer from disruptive interference caused by our own oculomotor behavior.

Similar mechanisms play a role at higher levels of perception. The high-level analog of blink suppression is the attentional blink; the analog of saccade suppression is change blindness. The origin of these phenomena in our perceptual system are currently under study. See, for instance, (Bowman and Wyble, 2007; Simione et al., 2012; Raffone et al., 2014) for an integrative treatment of a range of these phenomena in light of the architecture and dynamics of the visual/attentional system.

Content unity failure is also ignored in our maximal sense of self. Consider the persistent reasoning fallacies made famous by Kahneman and Tversky (1996). They clearly illustrate that people hold beliefs that are resistant against the logic they endorse. To distinguish these states from beliefs, which are normally subject to revision in face of contradictory evidence, Gendler (2008) has called them “aliefs”. According to Gendler, “to have an alief is, …, to have an innate or habitual propensity to respond to an apparent stimulus in a particular way. It is to be in a mental state that is (in a sense to be specified) associative, automatic and a rational. As a class, aliefs are states that we share with non-human animals; they are developmentally and conceptually antecedent to other cognitive attitudes that the creature may go on to develop. Typically, they are also affect laden and action-generating.” (p. 557). Examples are:a cinema-goer shrieking at a horror-movie; reluctance to throw darts at a picture of a loved one or for an avowed atheist to sign a pact with the devil. Aliefs, thus, are entrenched epistemical states that are to large extent immune against revision. Calling them entrenched suggests that their origins are buried in the evolution of the brain, even if the mechanisms may not be so easily identified.

According to Rosenthal (2001) and others, the psychological unity of consciousness is based on higher-order thought. Insofar thought involves reasoning rooted in belief, the various dissociations in ownership and content unity offer plenty of evidence that runs counter to this claim. We may, therefore, propose that insofar unity is a thought, it is not rooted in belief, but rather in something more akin to an alief. Aliefs are anchored in biology, and therefore resilient against the various disunities in the content of our everyday experience. This call for a biological explanation of the mechanisms that enable ownership unity and content disunity to co-exist, for which the notion of alief can only be a placeholder.

In sum, content unity is not required for unity in the minimal, nor in the maximal sense of self. This state of affairs is related to the biology of our brain. That does not mean the brain always lets people call the content of their experience their own. Schizophrenics, for instance, claim to hear voices. Presumably they mistake for other what is likely their own inner speech. Anosognosia (failure to perceive ones own disability), the different forms of unilateral neglect (failure to attend to one half field or one side of an object), and dementia (memory) are other pathologies where we may encounter violation of maximal ownership unity. All these syndroms clearly have a biological basis; for instance, brain lesions, even if the precise mechanisms that lead to the symptom are unknown. As for subtler symptoms, usually called “denial” or sometimes “repressed psychological states”, it is reasonable to speculate that these are due to milder forms of the biological mechanisms responsible for the clinical cases. Clearly, a deeper understanding of the brain is required to see what enables a healthy dialectic of ownership unity and content disunity, as well as the various syndroms that emerge when it goes wrong.

## Dynamical Representations

*If ever I to the moment shall say*:

Beautiful moment, do not pass away!

Then you may forge your chains to bind me

*—Faust, lines 1698–1701*

We need to understand, however, what enables the experience of unity and the absence of unity in experience to persist within a single individual. I propose that this is a property of brain activity and structure. Cognitive neuroscience has provided ample support for content disunity, by seeking to confirm hypotheses of functional specialization and localization in the brain. Localized modules seem to work independently and in parallel, only coupled through the signals that they pass through to each other through their synapses. Thus the scientific view seems slanted in favor of the conclusion that the experience of unity is illusory. However, I will argue that certain brain states occur at least intermittently that reveal a more global level of organization. These are the biological basis for the experience of unity.

What has led cognitive neuroscience to overlook these functions. It has at its disposal two well-established methods for studying brain activity: fMRI and EEG. Whereas the former has great spatial resolution, this is lacking in the second. Conversely, the first lacks the temporal resolution of the second. As a result, fMRI has been preferably used for studying the localization of function, while EEG for studying its temporal aspects. This has helped strengthen the belief that brain function can be studied in the spatial and temporal domains independently. This, researchers localize static sources in the brain with one method, and study the time course of their activity with the other, thinking this is all there is to the brain. However, this may be a mistake; important characteristics of the brain signal are lost when ignoring their spatiotemporal properties, such as traveling wave activity (Gong and van Leeuwen, 2009; Alexander et al., 2013; Bressloff, 2014).

Traveling waves are organizions that occur globally, at the scale of the whole head, both spontaneously during relaxed wakefulness (Ito et al., 2005) and evoked by external stimulation or self-initiated action (Alexander et al., 2013). Traveling waves provide spatiotemporal unity by passing through, and thus functionally connect, different local regions, modules, or circuits.

Traveling wave activity has an important role to play in neural signaling and communication, both at the circuit level (Gong and van Leeuwen, 2009) and at the level of EEG (see Alexander et al., 2013 for a review). Most importantly, it is associated with global workspace activity; in particular with global broadcasting and perceptual decision making (Nikolaev et al., 2010). Wave duration corresponds to estimates of the duration of the psychological present (Stroud, 1967). This may suggest that whole-head traveling waves are a mechanisms to effectuate and sustain the integration necessary for a minimal sense of self.

Wave activity that has been associated with consciousness is predominantly in the alpha and beta range, where alpha activity is associated with rest (Lehmann, 1990); beta activity with actively engaging the world (Nikolaev et al., 2010). Multiple different patterns of brain activity can give rise to the same type of event in experience, for instance the occurrence of a perceptual switching in ambiguous figures (Nakatani and van Leeuwen, 2006; Nakatani et al., 2012). Traveling waves might be necessary for experience of unity, they are by no means sufficient. Waves occur during deep sleep (Massimini et al., 2004), epilepsia, and prior to gestation; in other words, at times where we are typically not conscious. They are a biological phenomenon with several functions.

One of these functions could be relevant to the experienceof unity in the maximal sense. Broadly speaking, brain activity sustains the processes that together make up our mental life, cognition, and behavior. In order to do so in an intrinsically noisy system, brain activity needs to be geared towards stability; yet instability is also needed, in order for the brain to efficiently detach itself from a given process. This is why brain activity is on-going. On-going brain activity is poised between stable states (e.g., a global traveling wave) and periods of instability, where the activity shows more localized and transient patterns. This behavior is called itinerancy (Freeman, 2000; Kaneko and Tsuda, 2000). Such dynamics distinguishes the brain from devices like artificial neural networks; these are geared towards stable processes, from which only without external intervention can detach them.

Brains, basically, are networks of diffusively coupled nonlinear oscillators. In such systems, biological as well as artificial ones, itinerant regimes are pervasive. But these do not sustain any global activity pattern long enough to form the basis of our maximal sense of unity. However, their activity may support the evolution of a brain network structure that could do this job. Activity (function) and connectivity (structure) evolve in the brain on different time scales: fast activation dynamics leads to slow, adaptive modifications in connection strength (Skyrms and Pemantle, 2000) or structure (Gong and van Leeuwen, 2003, 2004; Zimmermann et al., 2004). This principle is re-iterated over multiple scales: there are multiple mechanisms of structural change operating on different time scales, and correspondingly, multiple time scales on which activity is coupled to these mechanisms.

Gong and van Leeuwen (2003, 2004) proposed an extremely simplified simple model in which ongoing, oscillatory activity has the role of establishing a certain type of network connectivity structure. The ongoing activation function leads to adaptive rewiring of network structure according to the Hebbian principle of “what fires together wires together”. The structure, in turn, helps sustain the function. The resulting symbiosis of structure and function plays an essential role in explaining how our development can manifest robust trends despite different individual life histories, and may help identify the conditions where it occasionally goes wrong.

**Figure 2:**
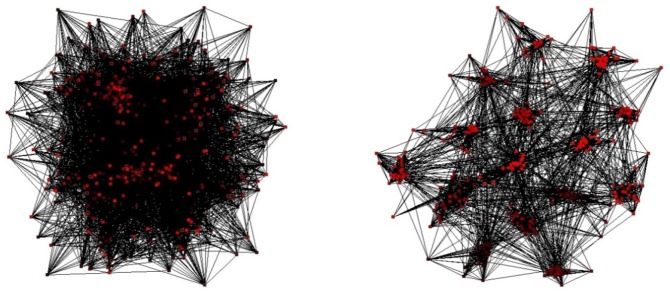
**A random network prior to (left) and after (right) several iterations of adaptive rewiring (From van Leeuwen, 2008)**.

The structures that emerge under the adaptive rewiring are universally of the “modular small world” type (see Figure 2). Small worlds are complex network structures that combine the advantages of a high degree of clustering, which characterizes regular networks, with the high degree of global connectedness observed in random-networks (Watts and Strogatz, 1998). Small world structures are optimally suitable for transfer of information within a network (Latora and Marchiori, 2001).

It is therefore not surprising that small-world structures feature in various domains of mental representation. The mental lexicon, for instance, was shown to have small-world structure, according to the graph of word co-occurrence in sentence contexts (Ferrer I Cancho and Solé, 2001). The properties of this structure influence the speed of retrieval from the mental lexicon during recognition (Chan and Vitevitch, 2009) and production of spoken words (Chan and Vitevitch, 2010), as well as long and short term memory retrieval (Vitevitch et al., 2012).

The existence of these structures runs counter to the influential claim by Fodor (1983) that cognitive systems cannot at the same time be informationally encapsulated (i.e., clustered) and globally well-connected and that, because of this, we must distinguish between encapsulated input and a cognitive, globally connected processing. Instead, the modular small-worlds category offers both, enabling an integrative view of, for instance, visual and cognitive processes.

Rather than perceptual and cognitive states being opposed to one another, as Fodor (1983) concept of modularity suggests, a small-world architecture of the brain would allow for different modes of activity within the same system: perceptual activity would correspond to regional waves confined to the clusters of the perceptual system and operating in parallel or as cascades; these could influence other clusters by global wave percolation. The percolation would meet with waves emerging from different sources and engage in at times collaborative, at times competitive interaction. Some regional activity may prevail and capture the system. This may explain, for instance, why a visual illusion may prevail, even though we know of its illusory character.

That brain connectivity has the characteristics of a small world is quite well-established (Sporns and Zwi, 2004; He et al., 2007; Bullmore and Sporns, 2009; Bullmore and Bassett, 2011; Sporns, 2011; Gallos et al., 2012). Absence of small-world structure in the brain is associated with brain pathologies such brain tumors (Bartolomei et al., 2006), epilepsy (Ponten et al., 2007) and, in particular, schizophrenia (Andreasen, 1999; Micheloyannis et al., 2006; Rubinov et al., 2009a) and Alzheimer’s disease (Stam, 2004; Stam et al., 2007; Frantzidis et al., 2014).

Already in its simplest version, the adaptive rewiring principle explains how some anomalies found in Alzheimer’s and schizophrenia patients may arise; in particular the counterintuitive observation that schizophrenic patients suffer from hyperglobality (Rubinov et al., 2009a). According to the model, this is because under adaptive rewiring, the property of clustering of connections is more vulnerable to aselective network lesioning than global connectivity (van den Berg et al., 2012).

Glazebrook and Wallace (2014), associate lesions to *global* connectivity with autism spectrum disorders. It is highly intriguing that some autism spectrum patients appear to share some of the characteristics with Kim Peek (and some other split-brain patients), even though clearly not autistic, including an seeming inability to deal with non-literal speech, prodigious attention to detail, and savant syndrome. Autism patients share with simultagnosia patients an inability to perceive and interpret the overall gestalt of a scene.

The mechanism these patients have in common may become clear if we take (re)wiring costs into account. Jarman et al. (2014) applied Gong and van Leeuwen’s adaptive rewiring approach, but built in a preference for local connections (as global connections are more costly). In this scheme, global connectivity is still formed, but the clusters that emerge are predominantly local. If the wiring costs are overweighted, the local clusters are hyper-regular. In the brain, such extreme modularity may reinforce local processes that interfere with the global communication. Interestingly, underweighting the wiring costs makes the clusters more diffuse; there are ectopic units in the clusters, something we observe in dyslexic patients.

The property of modularity in small world networks (Rubinov et al., 2009b) means the clusters interact via hubs. The hubs are specialized nodes or links that network evolution has given the role of mediating information transfer between communities. They synchronize, sometimes with one and sometimes with another subsystem, and can be considered as agents of change in the behavior of the regions to which they are connected.

The hubs and their interconnections may qualify as the architecture of the global workspace, whereas the modules they connect can be regarded as their client systems (see also Glazebrook and Wallace, 2014 for a similar proposal).

Here, I would therefore propose that the symbiosis of brain activity (traveling waves) and modular small-world architecture constitute the biological substrate for our maximum sense of self. Key is that the existence of different modules at various levels allows the system to keep pieces of content separate and relatively isolated from each other. The global connectivity implies that these discrete contents can at times be integrated. Our brains possess the conditions for both the disunity of conscious content, and the persistent ownership unity.

## Conclusions

As an antidote for the view that uor experience of unity is just a convenient fiction, I offered the hypothesis that its basis lies in the adaptive and dynamic mechanisms of the brain. I reached this view without taking a phenomenological stance, nor studying the brain from a first person perspective. I developed my view as a case for agnosticism with respect to “what-is-it-likeness”.

Agnosticism with respect to”what-is-it-likeness” does not constrain our efforts to study it. We may continue to address the question of what are the grounds for ascribing conscious experiences to ourselves, without presumptions about the validity of doing so. We may speak of phenomenal content in, for instance, a psychophysical, functional, or evolutionary perspective. We may study experience in relation to particular physical referents in the world (indirect psychophysics), in the brain (direct psychophysics), in relation to attention (Graziano, 2014), or as a product of optimization, learning, development, adaptation, and evolutionary processes. We may consider it a product of a global brain processes, such as cortico-cortical synchronization (Crick and Koch, 2001) or thalamo-cortical interactions (Edelman, 1992; Edelman et al., 2011). We may seek for the origins of consciousness in our biological evolution or that of our culture. We can treat conscious experience as causally efficacious within the context of these processes. For instance, having evolved the ability to understand ourselves as conscious beings, we were ready to express a moral understanding of our world, which further continues to shape our existence in our living environment. Thus, we may treat as given that we are living in a world endowed with meaning and value that ultimately rest on our phenomenal experience. None of this commits us to conscious experience as anything other than as a particularly stubborn belief, or alief.

But agnosticism does also invite us also to study certain biological mechanisms that could, at least in principle, support the validity of such beliefs, or aliefs. Some of these mechanisms are specific: saccade and blink suppression secure the continuity of our visual experience across perturbations from oculomotor behavior. Some are more generic: traveling waves produce a short-term unity in biological space-time as a pattern activity moves over of the cortical sheet. These patterns have their own, characteristic duration (Nikolaev et al., 2010) that corresponds, roughly, to the psychological present (Stroud, 1967) and hence they may be offered as a substrate for our minimal sense of self. Moreover, they may help in establishing and maintaining the architecture in the brain that can explain our maximal sense of self: the persistent experience of unity in face of the disunity of its content.

## Conflict of Interest Statement

The author declares that the research was conducted in the absence of any commercial or financial relationships that could be construed as a potential conflict of interest.

## Footnotes

^1^*The English translations of Faust quotes were found at http://en.wikiquote.org/wiki/Goethe%27s_Faust (downloaded 19-6-2014) or https://www.goodreads.com/work/quotes/16721-faust (downloaded 26-02-2015)*.

## References

[B1] Alexander D. M. Jurica P. Trengove C. Nikolalev A. R. Gepshtein S. Zvyagyntsev M.. (2013). Traveling waves and trial averaging: the nature of single-trial and averaged brain responses in large-scale cortical signals. Neuroimage 73, 95–112. 10.1016/j.neuroimage.2013.01.016 23353031

[B2] Andreasen N. C. (1999). A unitary model of schizophrenia. Bleuler’s “fragmented phrene” as schizencephaly. Arch. Gen. Psychiatry 56, 781–787. 10.1001/archpsyc.56.9.781 12884883

[B3] Anstis S. (2010). “Illusions of time, space and motion: flash-lag meets chopsticks and reversed phi,” in Space and Time in Perception and Action, eds Nijhawan R. Khurana B. (New York, NY: Cambridge University Press), 408–421

[B4] Baars B. (1988). A Cognitive Theory of Consciousness. New York, NY: Cambridge University Press.

[B5] Baars B. (1997). In the Theater of Consciousness: The Workspace of the Mind. New York, NY: Oxford University Press.

[B6] Bartolomei F. Bosma I. Klein M. Baayen J. C. Reijneveld J. C. Postma T. J.. (2006). Disturbed functional connectivity in brain tumour patients: evaluation by graph analysis of synchronization matrices. Clin. Neurophysiol. 117, 2039–2049. 10.1016/j.clinph.2006.05.018 16859985

[B7] Blanke O. Mentzinger T. (2009). Full-body illusiona and minimal phenomenal selfhood. Trends Cogn. Sci. 13, 7–13. 10.1016/j.tics.2008.10.003 19058991

[B8] Bowman H. Wyble B. (2007). The simultaneous type, serial token model of temporal attention and working memory. Psychol. Rev. 114, 38–70. 10.1037/0033-295X.114.1.38 17227181

[B9] Bressloff P. C. (2014). Waves in Neural Media: From Single Neurons to Neural Fields. Lecture Notes on Mathematical Modelling in the Life Sciences. New York, NY: Springer Verlag.

[B10] Bullmore E. T. Bassett D. S. (2011). Brain graphs: graphical models of the human connectome. Annu. Rev. Clin. Psychol. 7, 113–140. 10.1146/annurev-clinpsy-040510-143934 21128784

[B11] Bullmore E. Sporns O. (2009). Complex brain networks: graph theoretical analysis of structural and functional systems. Nat. Rev. Neurosci. 10, 186–198. 10.1038/nrn2618 19190637

[B88] Chalmers D. (1996). The Conscious Mind: In Search of a Fundamental Theory. New York, NY: Oxford University Press.

[B13] Chan K. Y. Vitevitch M. S. (2009). The influence of the phonological neighborhood clustering coefficient on spoken word recognition. J. Exp. Psychol. Hum. Percept. Perform. 35, 1934–1949. 10.1037/a0016902 19968444 PMC2791911

[B14] Chan K. Y. Vitevitch M. S. (2010). Network structure influences speech production. Cogn. Sci. 34, 685–697. 10.1111/j.1551-6709.2010.01100.x 21564230

[B15] Crick F. Koch C. (2001). “Consciousness and neuroscience,” in Philosophy and the Neurosciences, eds Bechtel W. Mundale P. Mandik J. Stufflebeam R. S. (Malden, MA: Blackwell), 254–277.

[B16] Dennett D. (1992). “The self as a center of narrative gravity,” in Self and Consciousness: Multiple Perspectives, eds Kessel F. Cole P. Johnson D. (Hillsdale, NJ: Erlbaum), 103–115.

[B17] Drummond S. R. Dutton G. N. (2007). Simultanagnosia following perinatal hypoxia: a possible pediatric variant of Balint syndrome. J. AAPOS 11, 497–498. 10.1016/j.jaapos.2007.03.007 17933675

[B18] Edelman G. M. (1992). Bright Air, Brilliant Fire: On the Matter of the Mind. New York, NY: Basic Books.

[B19] Edelman G. M. Gally J. A. Baars B. J. (2011). Biology of consciousness. Front. Psychol. 2:4. 10.3389/fpsyg.2011.00004 21713129 PMC3111444

[B21] Ferrer I Cancho R. Solé R. V. (2001). The small world of human language. Proc. Biol. Sci. 268, 2261–2265. 10.1098/rspb.2001.1800 11674874 PMC1088874

[B22] Fodor J. A. (1983). Modularity of Mind: An Essay on Faculty Psychology. Cambridge, MA: MIT Press.

[B23] Fodor J. A. (2000). The Mind Doesn’t Work That Way. The Scope and Limits of Computational Psychology. Cambridge, MA: MIT Press.

[B24] Frantzidis C. A. Vivas A. B. Klados M. A. Tsolaki M. Bamadis P. D. (2014). Functional disorganization of small-world brain networks in mild Alzheimer’s disease and amnestic mild cognitive impairment: an EEG study using relative wavelet entroy (RWE). Front. Aging Neurosci. 6:224. 10.3389/fnagi.2014.00224 25206333 PMC4144118

[B25] Freeman W. J. (2000). Neurodynamics. An Exploration of Mesoscopic Brain Dynamics. London, UK: Springer Verlag.

[B26] Gallos L. A. Makse H. A. Sigman M. (2012). A small world of weak ties provides optimal global integration of self-similar modules in functional brain networks. Proc. Natl. Acad. Sci. U S A 109, 2825–2830. 10.1073/pnas.1106612109 22308319 PMC3286928

[B27] Gazzaniga M. S. (1989). Organisation of the human brain. Science 245, 947–952. 10.1126/science.2672334 2672334

[B28] Gazzaniga M. S. Bogen J. E. Sperry R. W. (1962). Some functional effects of sectioning the cerebral commisures in man. Proc. Natl. Acad. Sci. U S A 48, 1765–1769. 10.1073/pnas.48.10.1765 13946939 PMC221037

[B29] Gendler T. S. (2008). Alief in action (and reaction). Mind Lang. 23, 552–585 10.1111/j.1468-0017.2008.00352.x

[B30] Georgalis N. (2006). Representation and the first-person perspective. Synthese 150, 281–325 10.1007/s11229-004-6268-5

[B31] Glazebrook J. F. Wallace R. (2014). Pathologies in functional connectivity, feedback control and robustness: a global workspace perspective on autism spectrum disorders. Cogn. Process. 16, 1–16. 10.1007/s10339-014-0636-y 25326271

[B32] Gong P. van Leeuwen C. (2003). Emergence of scale-free network with chaotic units. Physica A Stat. Mech. Appl. 321, 679–688 10.1016/s0378-4371(02)01735-1

[B33] Gong P. van Leeuwen C. (2004). Evolution to a small-world network with chaotic units. Europhys. Lett. 67, 328–333 10.1209/epl/i2003-10287-7

[B34] Gong P. van Leeuwen C. (2009). Distributed dynamical computation in neural circuits with propagating coherent activity patterns. PLoS Comput. Biol. 5:e1000611. 10.1371/journal.pcbi.1000611 20019807 PMC2787923

[B35] Graziano M. (2014). Are we really conscious? Available online at: http://www.nytimes.com/2014/10/12/opinion/sunday/are-we-really-conscious.html?smid=fb-shareand_r=0

[B36] He Y. Chen Z. J. Evans A. C. (2007). Small-world anatomical networks in the human brain revealed by cortical thickness from MRI. Cereb. Cortex 17, 2407–2419. 10.1093/cercor/bhl149 17204824

[B37] Hintikka J. (1962). Cogito ergo sum: inference or performance? Philos. Rev. 71, 3–32 10.2307/2183678

[B38] Ito J. Nikolaev A. R. van Leeuwen C. (2005). Spatial, temporal structure of phase synchronization of spontaneous EEG alpha activity. Biol. Cybern. 92, 54–60. 10.1007/s00422-004-0533-z 15650899

[B40] Jarman N. Trengove C. Steur E. Tyukin I. van Leeuwen C. (2014). Spatially constrained adaptive rewiring in cortical networks creates spatially modular small world architectures. Cogn. Neurodyn. 8, 479–497 10.1007/s11571-014-9288-y PMC4571644 26396647

[B41] Jurica P. Gepshtein S. Tyukin I. van Leeuwen C. (2013). Sensory optimization by stochastic tuning. Psychol. Rev. 120, 798–816. 10.1037/a0034192 24219849 PMC3877163

[B42] Kahneman D. Tversky A. (1996). On the reality of cognitive illusions. Psychol. Rev. 103, 582–591. 10.1037/0033-295x.103.3.582 8759048

[B43] Kaneko K. Tsuda I. (2000). Complex Systems, Chaos and Beyond: A Constructive Approach with Applications in Life Sciences. Berlin, Germany: Springer Verlag.

[B44] Kirk R. (1999). The inaugural address: why there couldn’t be zombies. Aristotalian Soc. Suppl. Vol. 73, 1–16 10.1111/1467-8349.00046

[B45] Kripke S. (1980). Naming and Necessity. Cambridge, MA: Harvard University Press.

[B46] Latora V. Marchiori M. (2001). Efficient behavior of small-world networks. Phys. Rev. Lett. 87:198701. 10.1103/physrevlett.87.198701 11690461

[B47] Lehmann D. (1990). “Brain electric microstates and cognition: the atoms of thought,” in Machinery of the Mind, ed John E. R. (Boston, MA: Birkhäuser), 209–224.

[B89] Loar B. (1990). Phenomenal states. Philos. Persp. 4, 81–108 10.2307/2214188

[B48] Massimini M. Huber R. Ferrarelli F. Hill S. Tononi G. (2004). The sleep slow oscillation as a traveling wave. J. Neurosci. 24, 6862–6870. 10.1523/jneurosci.1318-04.2004 15295020 PMC6729597

[B49] McCann J. J. Parraman C. Rizzi A. (2014). Reflectance, illumination and appearance in color constancy. Front. Psychol. 5:5. 10.3389/fpsyg.2014.00005 24478738 PMC3901009

[B51] Micheloyannis S. Pachou E. Stam C. J. Breakspear M. Bitsios P. Vourkas M.. (2006). Small-world networks and disturbed functional connectivity in schizophrenia. Schizophr. Res. 87, 60–66. 10.1016/j.schres.2006.06.028 16875801

[B52] Nagel T. (1974). What is it like to be a bat? Philos. Rev. 83, 835–850.

[B53] Nakatani H. Orlandi N. van Leeuwen C. (2012). Reversing as a dynamic process: variability of ocular and brain events in perceptual switching. J. Conscious. Stud. 19, 117–140.

[B54] Nakatani H. van Leeuwen C. (2006). Transient synchrony of distant brain areas and perceptual switching. Biol. Cybern. 94, 445–457. 10.1007/s00422-006-0057-9 16532332

[B55] Nikolaev A. R. Gepshtein S. Gong P. van Leeuwen C. (2010). Duration of coherence intervals in electrical brain activity in perceptual organization. Cereb. Cortex 20, 365–382. 10.1093/cercor/bhp107 19596712 PMC2803735

[B56] Perry B. D. (2002). Childhood experience and the expression of genetic potential: what childhood neglect tells us about nature and nurture. Brain Mind 3, 79–100 10.1023/A:1016557824657

[B57] Peterson M. A. Gibson B. S. (1991). Directing spatial attention within an object: altering the functional equivalence of shape descriptions. J. Exp. Psychol. Hum. Percept. Perform. 17, 170–182. 10.1037//0096-1523.17.1.170 1826310

[B58] Peterson M. A. Hochberg J. (1983). Opposed-set measurement procedure: a quantitative analysis of the role of local cues and intention in form perception. J. Exp. Psychol. Hum. Percept. Perform. 9, 183–193 10.1037//0096-1523.9.2.183

[B59] Ponten S. C. Bartolomei F. Stam C. J. (2007). Small-world networks and epilepsy: graph theoretical analysis of intracerebrally recorded mesial temporal lobe seizures. Clin. Neurophysiol. 118, 918–927. 10.1016/j.clinph.2006.12.002 17314065

[B60] Raffone A. Srinivasan N. van Leeuwen C. (2014). Perceptual awareness and its neural basis: bridging experimental and theoretical paradigms. Philos. Trans. R. Soc. Lond. B Biol. Sci. 369:20130203. 10.1098/rstb.2013.0203 24639576 PMC3965159

[B61] Ramachandran V. S. Blakeslee S. (1998). Phantoms in the Brain: Probing the Mysteries of the Human Mind. New York, NY: William Morrow.

[B62] Rosenthal D. M. (1986). Two concepts of consciousness. Philos. Stud. 49, 329–359 10.1007/bf00355521

[B63] Rosenthal D. M. (2001). Consciousness and Mind. Oxford, UK: Clarendon Press.

[B65] Rubinov M. Knock S. Stam C. J. Micheloyannis S. Harris A. Williams L.. (2009a). Small-world properties of nonlinear brain activity in schizophrenia. Hum. Brain Mapp. 30, 403–416. 10.1002/hbm.20517 18072237 PMC6871165

[B64] Rubinov M. Sporns O. van Leeuwen C. Breakspear M. (2009b). Symbiotic relationship between between brain structure and dynamics. BMC Neurosci. 10:55. 10.1186/1471-2202-10-55 19486538 PMC2700812

[B66] Schiffer F. Zaidel E. Bogen J. Chasan-Taber S. (1998). Different psychological status in the two hemispheres of two split-brain patients. Neuropsychiatry Neuropsychol. Behav. Neurol. 11, 151–156. 9742514

[B67] Schwitzgebel E. (2008). The unreliability of naive introspection. Philosophical Review 117, 245–273 10.1215/00318108-2007-037

[B68] Simione L. Raffone A. Wolters G. Salmas P. Nakatani C. Belardinelli M. O.. (2012). ViSA: a neurodynamic model for visuospatial working memory, attentional blink and conscious access. Psychol. Rev. 119, 745–769. 10.1037/a0029345.supp 22823385

[B69] Skyrms B. Pemantle R. (2000). A dynamic model of social network formation. Proc. Natl. Acad. Sci. U S A 97, 9340–9346. 10.1073/pnas.97.16.9340 10922082 PMC16869

[B70] Smullyan R. M. (1980/2002). An unfortunate dualist. from: this book needs no name, Reprinted in Philosophy of Mind, Classical and Contemporary Readings., ed Chalmers D. (New York, NY: Oxford University Press), 53–55.

[B71] Sporns O. (2011). The human connectome: a complex network. Ann. N Y Acad. Sci. 1224, 109–125. 10.1111/j.1749-6632.2010.05888.x 21251014

[B72] Sporns O. Zwi J. D. (2004). The small world of the cerebral cortex. Neuroinformatics 2, 145–162. 10.1385/ni:2:2:145 15319512

[B73] Stam C. J. (2004). Functional connectivity patterns of human magnetoencephalographic recordings: a ‘small-world’ network? Neurosci. Lett. 355, 25–28. 10.1016/j.neulet.2003.10.063 14729226

[B74] Stam C. J. Jones B. F. Nolte G. Breakspear M. Scheltens P. (2007). Small-world networks and functional connectivity in Alzheimer’s disease. Cereb. Cortex 17, 92–99. 10.1093/cercor/bhj127 16452642

[B75] Stroud J. M. (1967). The fine structure of psychological time. Ann. N Y Acad. Sci. 138, 623–631 10.1111/j.1749-6632.1967.tb55012.x

[B76] Teicher M. H. (2002). Scars that won’t heal: the neurobiology of child abuse. Sci. Am. 286, 68–75. 10.1038/scientificamerican0302-68 11857902

[B77] Treffert D. A. Christensen D. D. (2006). Inside the mind of a savant. Sci. Am. mind 17, 52–55 10.1038/scientificamericanmind0606-50 16323698

[B78] van den Berg D. Gong P. Breakspear M. van Leeuwen C. (2012). Fragmentation: loss of global coherence or breakdown of modularity in functional brain architecture? Front. Syst. Neurosci. 6:20. 10.3389/fnsys.2012.00020 22479239 PMC3316147

[B79] van Leeuwen C. (2007). What needs to emerge to make you conscious? J. Conscious. Stud. 14, 115–136.

[B80] van Leeuwen C. (2008). Chaos breeds autonomy: connectionist design between bias and babysitting. Cogn. Process 9, 83–92. 10.1007/s10339-007-0193-8 17924155

[B81] van Leeuwen C. Smit D. J. A. (2012). Restless brains, wandering minds, in Being in Time: Dynamical Models of Phenomenal Awareness. Advances in Consciousness Research, eds Edelman S. Fekete T. Zach N. (Amsterdam, NL: John Benjamins PC), 121–147.

[B82] Vitevitch M. S. Chan K. Y. Roodenrys S. (2012). Complex network structure influences processing in long-term and short-term memory. J. Mem. Lang. 67, 30–44. 10.1016/j.jml.2012.02.008 22745522 PMC3381451

[B83] Watts D. Strogatz S. (1998). Collective dynamics of ‘small-world’ networks. Nature 393, 440–442. 9623998 10.1038/30918

[B84] Welshon R. (2013). Searching for the neural realizers of ownership unity. Philos. Psychol. 26, 839—862 10.1080/09515089.2012.713164

[B86] Wundt W. Pintner R. (1912). Consciousness and attention, in An Introduction to Psychology, eds Wundt W. Pintner R. (New York, NY: MacMillan Co), 1–42).

[B87] Zimmermann M. G. Eguıluz V. M. Miguel M. S. (2004). Coevolution of dynamical states and interactions in dynamic networks. Phys. Rev. E Stat. Nonlin. Soft. Matter Phys. 69:065102R. 10.1103/physreve.69.065102 15244650

